# Acyclovir extravasation in a newborn: a case report

**DOI:** 10.1186/s13256-024-04585-1

**Published:** 2024-06-07

**Authors:** Shirin Shamel, Mohammad Reza Zarkesh

**Affiliations:** 1 Department of Neonatology, Yas Hospital Complex, Tehran University of Medical Sciences, Sarv Ave., North Nejatolahi Street, Tehran, 1598718311 Iran; 2 Maternal, Fetal, and Neonatal Research Center, Family Health Research Institute, Tehran University of Medical Sciences, Tehran, Iran

**Keywords:** Acyclovir, Extravasation, Neonate

## Abstract

**Objective:**

Extravasation of infused drugs is not a rare problem in medical practice. Acyclovir is a vesicant and an antiviral medication commonly used for young children. In the present study, we presented a neonate with soft tissue damage due to acyclovir extravasation.

**Case report:**

A female newborn (Iranian, Asian) with gestational age 37^+2^ weeks and breech presentation was born by Cesarean delivery from a mother with a recent history of Herpes simplex virus (HSV) infection (Yas Women’s Hospital, Tehran, Iran). Intravenous administration of acyclovir was initiated through a peripheral catheter inserted on the dorsal side of the left hand. A few minutes after the second dose, the patient showed a diffused firm swelling, local discoloration, and induration in the dorsum of the hand. The peripheral catheter was removed immediately. Hyaluronidase was injected subcutaneously in five different regions around the catheterization site. Intermittent limb elevation and cold compression (for 10 minutes) were applied. Serial follow-ups and examinations were performed hourly to check limb inflammation, ischemia, and compartment syndrome. The limb swelling and discoloration significantly improved 4 hours after the second dose of hyaluronidase.

**Conclusion:**

Early diagnosis of acyclovir extravasation and immediate management could prevent severe complications in neonates. Further studies are needed to suggest a standard approach and treatment protocol for acyclovir extravasation.

## Introduction

Extravasation of intravenously infused drugs refers to the leakage of vesicant intravenous medical solutions from blood vessels to the surrounding tissues [1]. The frequency of this complication among adult subjects is reported to be 0.1–6.5% [2]; however, this frequency in children and neonates has not been reported precisely due to a lack of enough documented data [3]. A few local articles have reported an incidence of 2–46% in hospitalized neonates and children [4–7].

Children, particularly neonates, are at greater risk of extravasation due to difficulties in catheter fixation through their tiny and fragile vessels, thin subcutaneous fat, and limited ability to report pain [1, 3]. Manifestations of extravasation are usually minor and resolvable. Local swelling, erythema, and pruritus are the common presentations, while a few cases showed severe degrees of blistering, discomfort, and numbness, ending up with persistent tissue necrosis [1, 8].

The risk factors of extravasation are classified into the patient, procedure, and equipment-related factors. Patient-related factors include tiny fragile veins, easily-burst vessels during catheterization, highly-moving extremities, and difficulties in peripheral venous access in obese subjects and children. The most prevalent procedure-related factor is a lack of experience in catheterization resulting in vein puncture attempts. The equipment-related elements are the implementation of inappropriate catheters, needles, or poor fixation of intravenous cannulas [1].

Herpes simplex virus (HSV) is responsible for a viral infection causing mortality in 60% of untreated newborns [9]. Acyclovir, as an acyclic synthetic analogue of guanosine, inhibits HSV-DNA replication. Acyclovir is the first-line medication for HSV infection, preventing disease dissemination and nervous system involvement [10].

To our knowledge, four cases of acyclovir extravasation have been reported, and all of them were adults [2, 8, 11, 12]. The present study, as the first investigation, presents a neonate with acyclovir extravasation and discusses the different issues of its treatment.

## Case presentation

A female newborn (Iranian, Asian) with gestational age 37^+2^ weeks and breech presentation was born by Cesarean delivery on 2022-June-09 at 4:00 PM. Regarding the obstetric history, an ultrasound examination at 32 weeks of gestation showed an intrauterine growth restriction (IUGR) fetus with normal color Doppler study. Her mother, at admission time, reported a recent history of primary HSV infection (first episode) with typical active and visible genital lesions (2 weeks ago). After delivery, the neonate had unremarkable findings related to the general physical exam and reflexes. No abnormal skin or eye lesions were found as well. Neonate’s birth weight was 2190 g. Apgar scores at minutes 1 and 5 were 9 and 10, respectively.

Due to a positive history of maternal HSV infection, an emergent consultation with a Subspecialist of Pediatric Infectious Disease was performed.

Initiation and continuation of intravenous acyclovir were performed because of the potential early course of the disease and the high possibility of false negative HSV PCR in infants [8, 13]. Acyclovir administration (60 mg/kg/day diluting in normal saline 0.9% divided into 3 doses) with close observation was ordered. Consequently, blood and cerebrospinal fluid (CSF) samples were collected and sent to the laboratory for analysis. Laboratory tests were cell blood count, arterial and cord blood gas analyses, liver function test, HSV blood PCR, HSV blood culture, HSV surface culture (conjunctivae, mouth, and nasopharynx), as well as CSF study (cell count, glucose, protein, and HSV PCR). The results are shown in Table 1. Brain and abdominal ultrasound examinations were also done. Ultrasound study showed no remarkable findings.

**Table 1:** The results of laboratory blood, arterial gas, and cerebrospinal fluid tests before and after acyclovir administration

Factors	Results	
	Before	After
*Arterial blood gas results*		
PH	7.34	7.38
PaCO_2_ (mmHg)	26.2	34.7
PaO_2_ (mmHg)	78.9	82.9
HCO_3_ (meq/L)	13.9	19.2
Base excess (mEq/L)	− 9.7	− 5.3
*Umbilical cord blood gas results*		–
PH	7.38	
PaCO_2_ (mmHg)	30.7	
PaO_2_ (mmHg)	17.6	
HCO_3_ (meq/L)	13.9	
Base excess (mEq/L)	− 6.2	
*Blood test*		
Hb (g/dL)	15.9	14.6
HCT (%)	45.2	43.5
Platelet (cells/mcL)	297,000	303,000
MCV (fL)	106.86	104.7
MCH	37.59	37.61
MCHC	35.18	36.1
RBC (10^6^/mm^3^)	4.23	4.21
WBC (cells/mcL)	12700H	14600H
RDW CV	17.1	16.3
Neutrophils (cells/mcL)	45	56
Lymphocytes (cells/mcL)	47	42
Mixed	8	2
Bilirubin total	9.6	8.5
Bilirubin direct	0.7	0.5
Na (mmol/L)	135	137
K (mmol/L)	3.8	4.5
Mg (mEq/L)	1.8	1.6
Ca (mg/dL)	10.8	9.6
Retic count (%)	3.3	0.2
Peripheral blood smear	Normal	–
G6PD	Sufficient	–
AST	29	–
ALT	11	–
CRP	0.1	–
Blood group	O positive	–
Coombs direct	Negative	–
Blood sugar	70	–
TSH (mIU/L)	–	2.7
FT4 (pmol/L)	–	0.9
Eye culture	No growth after 72 hours	–
Blood culture	Negative	–
HSV PCR quantitative	Non-reactive	–
*CSF analysis*		–
RBC	0	
WBC	0	
Protein	70	
Glucose	60	
CSF culture	Negative	
Smear for bacteria	Negative	

**Fig. 1:**
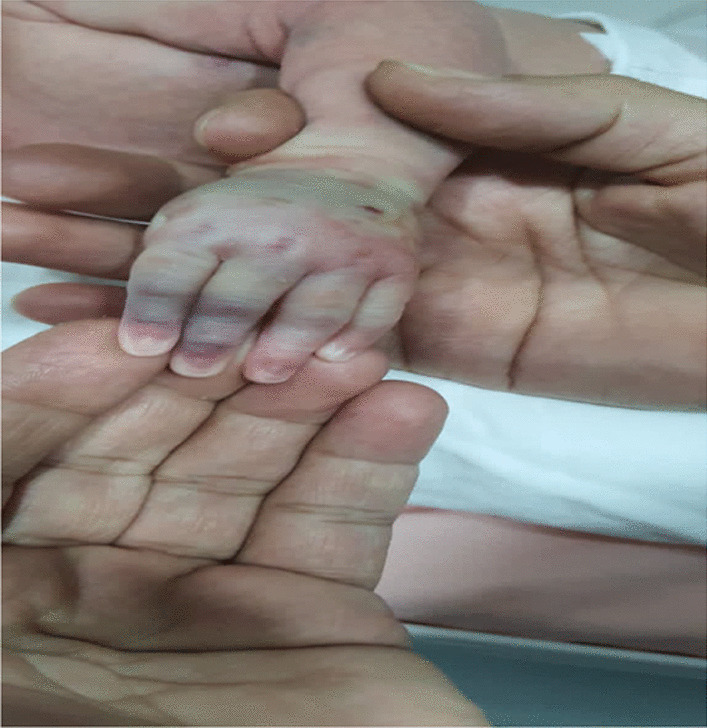
Local manifestations of acyclovir extravasation

**Fig. 2:**
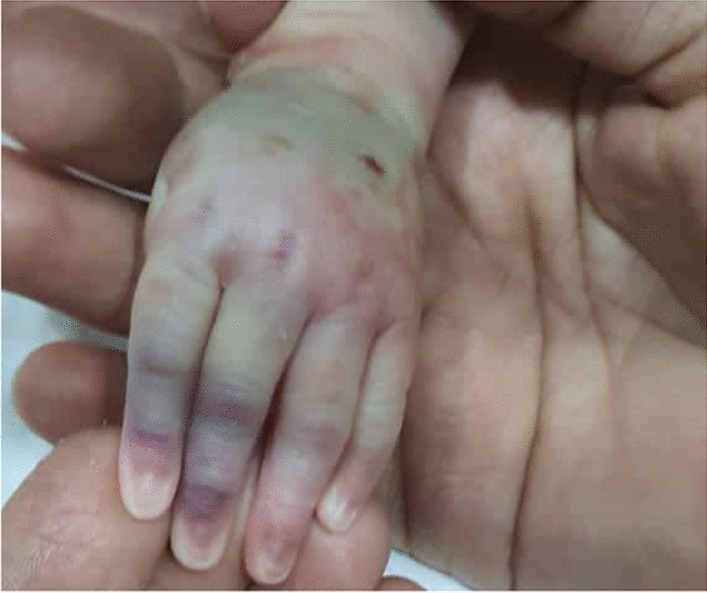
Local manifestations of acyclovir extravasation

**Fig. 3:**
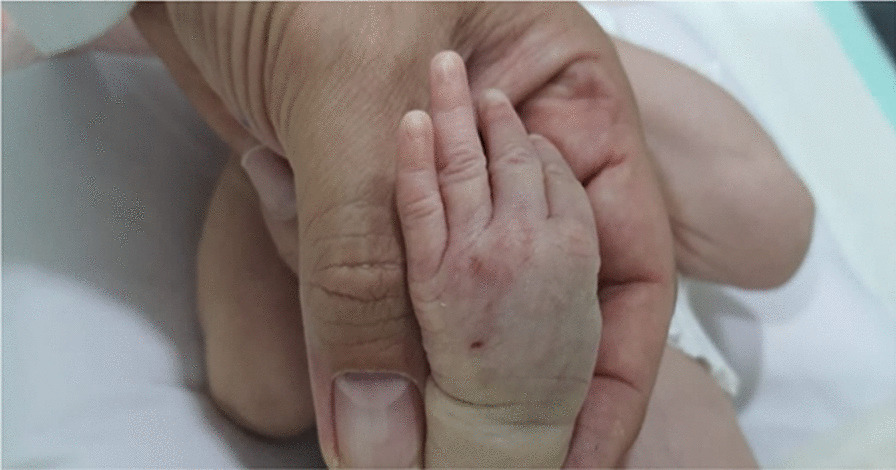
Improvement of limb swelling and discolouration after intervention

At 6:00 PM., intravenous administration of Acyclovir was initiated through a peripheral catheter inserted on the dorsal side of the left hand. At 2:00 AM., the second dose of Acyclovir was administered. About 3 minutes after drug infusion, the patient showed local manifestations of Acyclovir extravasation (Figs. 1 and 2). Physical examination demonstrated a diffused firm swelling, discoloration, and induration in the dorsum of the hand, which was compressible. Passive flexion and extension of the fingers were intact. Distal pulses and capillary refilling exams were also normal. The peripheral catheter was removed immediately. Then, consultations with a Neonatologist and Pediatric Dermatologist were performed. Accordingly, over the next 5 minutes after discontinuing the Acyclovir infusion, Hyaluronidase (100 units/cc of normal saline) was injected subcutaneously in five different regions around the catheterization site. The same dose was repeated 6 hours later. Serial follow-up and examination were performed hourly to check limb inflammation, ischemia, and compartment syndrome. Vital signs were also monitored frequently. Intermittent limb elevation and cold compression (for 10 minutes) were applied every 6 hours. The limb swelling and discoloration significantly improved 4 hours after the second dose of hyaluronidase (Fig. 3). No notable side effect was also observed after hyaluronidase administration.

The resumption of HSV treatment with acyclovir was done through the insertion of another peripheral venous access. Laboratory examination was also repeated after acyclovir infusion (Table 1). Findings related to laboratory results on the 5th day showed that quantitative HSV blood PCR, eye culture, blood culture, surface cultures, and surface HSV PCR were all negative. All measured indices by CSF study were within normal ranges. The neonate remained asymptomatic in terms of probable HSV manifestations during the admission period.

The asymptomatic case with unremarkable laboratory results was discharged on the 6th day. Forty-eight hours after discharge, she was visited at the outpatient department. The signs of extravasation completely disappeared after a month. Moreover, orthopedic and hematologic follow-up examinations revealed no remained sequelae. The sequence of all events and interventions is shown in Table 2.

**Table 2:** The sequence of events and interventions

	Measures	Time
1	Cesarean delivery	4:00 PM
2	Brain and abdominal ultrasound examinations were also done	5:00 PM
3	Blood and cerebrospinal fluid sampling	5:45 PM
4	Intravenous administration of acyclovir was initiated (first dose)	6:00 PM
5	Administration of the second dose	2:00 AM (8 hours after the first dose)
6	Local manifestations of acyclovir extravasation	2:03 AM
7	The peripheral catheter was removed immediately	2:04 AM
8	Initiation of hyaluronidase	2:10 AM
9	The same dose of hyaluronidase was repeated	8:10 AM
10	Neonate was discharged	On 6th day
11	Follow-up visit	On 8th day

## Discussion

In the present study, we reported acyclovir extravasation and successful management of related symptoms. Although few studies presented this complication in adults, our study is the first investigation presenting a neonate case. Due to the lack of such studies in the field of pediatrics, there is no specific protocol for the treatment of extravasation in neonates. Our case presentation demonstrates that side effects of acyclovir extravasation and early management should be a consideration.

Manifestations in our case were early-onset; however, due to timely, efficient, and effective medical interventions were not potentially severe. As young children, particularly neonates are unable to report pain or discomfort sensations related to drug extravasation, medical staff by close observation, should be aware of any discoloration, erythema, swelling, induration, or other inflammatory responses around the site of catheterization. Drugs concerning extravasation are classified into three vesicant, irritant, and non-vesicant subgroups [1]. Acyclovir, the commonly used antiviral medication is a vesicant drug [8, 14], therefore medical cautions should be highly considered. The osmolality of Acyclovir (278 mOsm/kg) is close to the plasma’s, and its toxicity relates to high alkalinity (pH = 11) [2, 14]. These properties are responsible for chemical inflammation and tissues damage in case of extravasation. It should be noted that patients with late-onset manifestations or unspecific signs need more attention [8]. Other risks related to intravenous fluid therapy, like stopping or slowing down fluid infusion, blood backflow, or leakage of the drug around the needle, also should raise doubt regarding the extravasation process [1]. In addition, alkaline property of acyclovir, using solutions with extreme basic pH (> 9) can increase the risks of leakage and tissue damage.

In line with our observation, Sarıca *et al.* reported a case without any severe complication related to acyclovir extravasation because of close observation and tight management [11]. On the other hand, Neocleous *et al.* demonstrated a case with acyclovir extravasation that ended up with a residual scar secondary to tissue necrosis [8]. De Souza *et al.* also presented acyclovir extravasation in an adult diabetic case with severe edema in the hand and stiffness of the metacarpophalangeal joint due to fibrosis and lymphatic obstruction [12]. It was confirmed that any delayed or inadequate interventions in a such emergency might lead to severe problems like persistent lymphedema, full-thickness skin loss, tissue fibrosis, tendon necrosis, undesirable scars, or amputation [1, 8].

A previous study showed that the frequency of acyclovir extravasation is not rare among adult subjects (16%). It speculates that this complication would be more prevalent among neonates with fragile vessels and difficulties in catheterization. Hence, this diagnosis was suspected immediately in our case, and early medical interventions, including the stopping of drug infusion, removing the catheter, cold compress, and elevating the limb, could gradually recover the local signs. Another study also emphasized rapid nursing interventions after any suspicion of acyclovir extravasation. Discontinuation of intravenous infusion or injection and complete disconnection of the venous tube from the cannula were reported as the first line of intervention. Moreover, cannula removal after aspiration of as much extravasated drug as possible could be another beneficial intervention [15]. Manual pressure over the areas has not been suggested. Determination of complicated area with a pen and precise documentation has been shown as a helpful step in follow-up process. Limb elevation and thermal application (ice packs) were also reported as the following mandatory steps to decrease the capillary hydrostatic pressure and limit drug reabsorption/dispersion [1, 15]. Local warming was recommended for DNA-binding vesicant drug extravasation, but the choice of thermal application (cooling or warming) depends on the physician’s notification based on drug classification. A regular schedule for warming and cooling applications (10–15 minutes every 4 hours for 24–48 hours) has been suggested [1, 15].

We also administered hyaluronidase as an effective medication. Hyaluronidase is an enzyme that temporarily reduces the hyaluronic acid viscosity and also decreases the consistency of the intercellular tissues. This FDA (Food and Drug Administration) approved drug with unremarkable (0.05–0.1%) side effects has been recommended for the management of extravasations [16]. Administration of hyaluronidase should apply at the correct dosage and time. Prescription of subcutaneous or intradermal injection within an hour after extravasation could be helpful [2, 10]. Regarding the proper dosage, several factors like the amount of extravasated vesicant, the extent of subcutaneous damage, the results of physical examination, and the affected limbs are determinants [2, 17]. Although conservative management and hyaluronidase administration are suggested approaches for children and neonates, further clinical trials are needed [18]. Finally, the prescription of antidotes in vesicant extravasations is controversial and depends on the physician’s clinical view [2].

As a result, acyclovir extravasation requires prompt recognition and management by healthcare givers. Patient evaluation and examination would guide the healthcare team in decision-making regarding interventional or observational management. Close monitoring of the affected limb for any worsening signs is mandatory. The patient and the guardians (for children) should be warned against probable surgical interventions. Moreover, they should be informed about follow-up visits to prevent possible late complications such as persistent lymphatic edema and cellulitis.

## Conclusion

The present study highlights the potential risks related to acyclovir extravasation in neonates. Early diagnosis, immediate management, and follow-up visits could optimize the adverse outcome. Further studies are needed to suggest a standard approach and treatment protocol.

## Data Availability

The datasets related to our study are available from the corresponding author upon reasonable request.

## Abbreviations

HSV: Herpes simplex virus
CSF: Cerebrospinal fluid
PCR: Polymerase chain reaction
